# A systematic literature review of existing conceptualisation and measurement of mental health literacy in adolescent research: current challenges and inconsistencies

**DOI:** 10.1186/s12889-020-08734-1

**Published:** 2020-05-01

**Authors:** Rosie Mansfield, Praveetha Patalay, Neil Humphrey

**Affiliations:** 1 Institute of Education, University of Manchester, Ellen Wilkinson Building, M13 9PL, Manchester, UK; 2 Institute of Education and Faculty of Population Health Sciences, University College London, WC1E 6BT, London, UK

**Keywords:** Adolescent, Mental health literacy, Systematic literature review, Conceptualisation, Measurement

## Abstract

**Background:**

With an increased political interest in school-based mental health education, the dominant understanding and measurement of mental health literacy (MHL) in adolescent research should be critically appraised. This systematic literature review aimed to investigate the conceptualisation and measurement of MHL in adolescent research and the extent of methodological homogeneity in the field for meta-analyses.

**Methods:**

Databases (PsycINFO, EMBASE, MEDLINE, ASSIA and ERIC) and grey literature were searched (1997–2017). Included articles used the term ‘mental health literacy’ and presented self-report data for at least one MHL domain with an adolescent sample (10–19 years). Definitions, methodological and contextual data were extracted and synthesised.

**Results:**

Ninety-one articles were identified. There was evidence of conceptual confusion, methodological inconsistency and a lack of measures developed and psychometrically tested with adolescents. The most commonly assessed domains were mental illness stigma and help-seeking beliefs; however, frequency of assessment varied by definition usage and study design. Recognition and knowledge of mental illnesses were assessed more frequently than help-seeking knowledge. A mental-ill health approach continues to dominate the field, with few articles assessing knowledge of mental health promotion.

**Conclusions:**

MHL research with adolescent samples is increasing. Results suggest that a better understanding of what MHL means for this population is needed in order to develop reliable, valid and feasible adolescent measures, and explore mechanisms for change in improving adolescent mental health. We recommend a move away from ‘mental disorder literacy’ and towards critical ‘mental health literacy’. Future MHL research should apply integrated, culturally sensitive models of health literacy that account for life stage and acknowledge the interaction between individuals’ ability and social and contextual demands.

## Background

Around 50% of mental health difficulties have their first onset by age 15 [1, 2] and are associated with negative outcomes such as lower educational attainment and physical health problems [3]. Approximately 10–20% of young people are affected worldwide, and many more will experience impairing mental distress at varying degrees across the mental health continuum [4–8]. Adolescence is a critical period of transition, characterised by physical, cognitive, emotional, social and behavioural development [9]. It has therefore been identified as a particularly important developmental phase for improving ‘mental health literacy’ (MHL) and promoting access to mental health services [10, 11]. However, better understanding of the conceptualisation and measurement of MHL in this population is needed.

MHL was first defined as ‘*knowledge and beliefs about mental disorders which aid their recognition, management or prevention’* ( [12] pp 182) and consisted of six domains: *‘1) the ability to recognise specific disorders or different types of psychological distress; 2) knowledge and beliefs about risk factors and causes; 3) knowledge and beliefs about self-help interventions; 4) knowledge and beliefs about professional help available; 5) attitudes which facilitate recognition and appropriate help-seeking, and 6) knowledge of how to seek mental health information’* ( [13] pp 396). Domains were later revised to include early recognition, prevention and mental health first aid skills [14]. The most recent definition comprises four broad domains aligned with current definitions of health literacy: *‘1) understanding how to obtain and maintain positive mental health; 2) understanding mental disorders and their treatments; 3) decreasing stigma related to mental disorders, and 4) enhancing help-seeking efficacy (knowing when and where to seek help and developing competencies designed to improve one’s mental health care and self-management capabilities’* ( [15] pp 155).

In a review of MHL measurement tools, O’Connor et al. revealed that the most commonly assessed domain was recognition of mental disorders. No studies assessed either knowledge of how to seek information or knowledge of self-help interventions [16]. The focus on recognition of mental disorders, along with knowledge about risk factors, causes and appropriate treatments, has been criticised for promoting the psychiatric and biogenetic conceptualisation of mental illness [17, 18]. Despite being found to reduce blame, biogenetic explanations and attributions can lead to misconceptions about dangerousness and unpredictability and pessimism about recovery [19]. Early research also suggested that biogenetic causal theories increase a desire for social distance [20, 21]. MHL modelled on recognition of psychiatric labels, and diagnostic language such as ‘disorder’, often leads to psychosocial predictors being ignored, and more negative attitudes towards individuals experiencing mental distress [22, 23].

These criticisms, in line with broader socio-cultural approaches to literacy [24] understand MHL as a socio-political practice used to communicate, and make dominant, the psychiatric discourse. This appears to undermine attempts to reduce stigma, the most common outcome of school-based MHL interventions [25]. In their review of MHL measurement tools, O’Connor et al. excluded all disorder specific scales, claiming that ‘*MHL by definition should encompass knowledge and attitudes relating to a range of mental health disorders and concepts*.’ ( [16] pp 199). Chambers et al. further criticised current MHL definitions for being narrow in focus with a predominantly mental-ill health approach, ignoring the complete mental health state that goes beyond the dichotomy of illness and wellness [26, 27]. The difference between literacy about mental disorders and the ability to seek out, comprehend, appraise and apply information relating to the complete mental health state is an emerging point of discussion, and has seen MHL re-defined to include self-acquired knowledge and skills relating to positive psychology [28, 29]. This aligns with the World Health Organisation’s (WHO) definition of mental health, which includes subjective wellbeing, optimal functioning and coping, and recognises mental health beyond the absence of disorder [30].

In response to increasingly inclusive definitions of MHL, Spiker and Hammer presented the argument for MHL as a *‘multi-construct theory, rather than a multi-dimensional construct’* ( [31] pp 3). The proposal suggested that by stretching the MHL construct, researchers have reduced the consistent use of the definition across studies, resulting in heterogeneous measurement [32]. Reviews of the psychometric properties of MHL measurement tools support this argument, and conclude that more consistent measurement with valid scales is needed [33–36]. Spiker and Hammer also outline problems with construct irrelevant variance [31], in which measures capture more than they intended to. Furthermore, they note that construct proliferation or the ‘jingle jangle fallacy’ [37], in which scales may have different labels but measure the same construct, and vice versa, increase problems with discriminant validity. Understanding MHL as a multi-construct theory could help delineate between its broad domains: recognition, knowledge, stigma and help-seeking beliefs, and acknowledge their complexity.

Internationally, there is growing political interest in child and adolescent mental health promotion and education [6, 38]. Despite limited evidence, it is suggested that educating the public by improving their ability to recognise mental disorders, and increasing help-seeking knowledge, can promote population mental health [39, 40]. Furthermore, a reduction in stigmatising attitudes is consistently reported to improve help-seeking [41, 42]. MHL, by definition, includes these interacting domains. However, despite a comprehensive set of reviews that assess the psychometric properties of MHL measurement tools [33–36], there is no systematic literature review, to date, that assesses the current conceptualisation and measurement of MHL across adolescent research. Being able to clearly operationalise what is meant by a MHL intervention and meta-analyse their effectiveness, will have implications for the investment in school and population level initiatives. Similarly, being able to conduct time trend analyses that plot possible improvements in adolescents’ MHL against mental health outcomes, will reveal the extent to which population level improvements in MHL promote mental health. First though, we must have a clear picture of the understanding of MHL in adolescent research and how it is currently being measured.

### Objectives and research questions

The aim of the current study was therefore to examine the ways in which MHL has been conceptualised and measured in adolescent research to date, and explore the extent of methodological homogeneity in the field for meta-analyses. We set out to answer the following research questions: 1) What are the most common study designs, contexts, and aims? 2) How is MHL conceptualised? 3) What are the most commonly measured domains of MHL, and do these vary by study design and definition usage? 4) To what extent do articles use measures that have evidence of validity for use with adolescent samples? 5) Is there enough methodological homogeneity in the field to conduct meta-analyses?

## Method

A protocol was published on PROSPERO in December 2017 (reference: CRD42017082021), and was updated periodically to reflect the progress of the review. Relevant PRISMA guidelines for reporting were followed [43].

### Eligibility criteria

Articles were included with adolescent samples aged between 10 and 19 [44]. Samples with a mean age outside of this range were excluded. If no mean was presented and the age range fell outside of the criterion, articles were only included if results were presented for sub-groups (e.g. 12–17 years from a sample aged 12–25). General MHL and diagnosis-specific literacy research was included. Articles with quantitative study designs and extractable self-report data for at least one time point measurement of any MHL domain were eligible. These criteria ensured that only articles with extractable data from adolescents, who had not yet received any form of intervention were included. At the full text screening phase, articles published before 1997, based on the date of the first MHL definition [12], and those that did not explicitly use the term ‘mental health literacy’ or a diagnosis-specific equivalent (e.g. ‘depression literacy’) were excluded. By applying this criterion, the current study was able to present the number of articles that measured domains without referring to MHL. Identifying cases where researchers measure the same construct but use different labels is important when considering conceptualisation and meta-analyses.

Only articles available in English were included. Specific populations such as clinical/patient populations and juvenile offenders were excluded, as were university students. In contrast to schools in most countries, universities are not universal, with only a sub-set of young people entering higher education. University samples were therefore not seen as representative and often included participants outside the age criterion. Post-partum and later life neurocognitive disorders (e.g. Alzheimer’s disease) were removed given their limited relevance for this age group. In line with other MHL reviews [33], articles with a focus on substance abuse were excluded to avoid reviewing a large number of adolescent risk behaviour studies and substance abuse prevention programmes.

### Search strategy

The search strategy was developed to include a number of combinations of terms to ensure that literature relating to different domains of MHL were captured. Population terms such as ‘adolescen*’ or ‘young people*’ had to be present and mental health related terms (e.g. ‘mental health’ and ‘mental disorders’) were exploded to capture general MHL and diagnosis-specific studies. Similarly, outcome terms (e.g. ‘health literacy’ and ‘health education’) were exploded, and domain specific terms included (e.g. ‘knowledge’, ‘recogni*’, ‘attitud*’, ‘stigma*’, ‘help-seek*’, ‘prevent*’ or ‘positive*’). See Additional File 1. for an example search strategy.

### Data sources

The following databases were searched from their start date to the search dates (November 2017): PsycINFO, EMBASE, MEDLINE, ASSIA, and ERIC. Key authors were also contacted to identify grey literature. References were harvested from related reviews and all papers identified in the search. Hand searches of key authors’ publication lists were also conducted, and Google Scholar was used to find studies known by the authors but not identified in the database searches.

### Article selection

Results from the database searches were saved to Endnote and duplicates were removed. The lead author screened the article titles and abstracts to identify those that met the inclusion criteria. Full texts were then screened and reasons for exclusion were recorded. Any uncertainties were resolved through discussion with other members of the research team. A sub-set of 20 articles were screened at full text stage by the third author, and a strong level of agreement was found (k = .78, *p* = .001).

### Data extraction

Research was assessed on an article level (rather than by study) for the purposes of investigating the conceptualisation of MHL. The fact that authors break MHL down into component parts to write separate articles is support for identifying which domains are more commonly associated with the use of the term. Data on the following methodological factors were extracted from eligible articles using a uniform data extraction form: year of publication, country and setting (community (research conducted outside of the school setting e.g. population level surveys) vs. school-based research), study design (intervention vs. population-based), primary aims, MHL definition and use of the term, general MHL vs. diagnosis-specific literacy, number/types of MHL domains measured, and measurement tools (e.g. vignette, yes/no, Likert scales).

### Data analysis

**Fig. 1:**
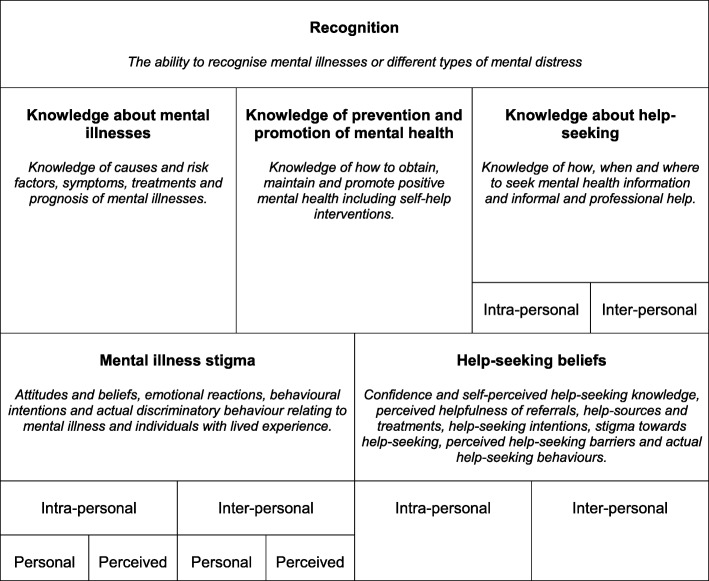
MHL Coding Framework

A content analysis was conducted using NVivo 12 to organise articles by their primary aim and understand the conceptualisation of MHL based on the definition presented and use of the term. Frequencies and percentages for each group were calculated and articles coded based on whether they included items related to general MHL or diagnosis-specific literacy. Existing definitions of MHL [12–15, 28] were used to create a coding framework that clearly delineated its broad constituent domains (e.g. recognition, knowledge, stigma and beliefs), the object of these domains (e.g. mental illnesses, mental health prevention and promotion, and help-seeking), and their directionality (e.g. self vs. other) – see Fig. 1.

Mental illness stigma was assessed using existing conceptualisation i.e. personal and perceived stigma relating to self (intra-personal) and others (inter-personal), and broad domains (e.g. attitudes and beliefs, emotional reactions, and social distancing) [45]. The coding of help-seeking beliefs was informed by the theory of planned behaviour [46], assessing not only help-seeking intentions but also help-seeking confidence and self-perceived help-seeking knowledge, perceived helpfulness of referrals, help-sources and treatments, help-seeking stigma and perceived help-seeking barriers. A distinction was also made between help-seeking beliefs for self (intra-personal) vs. others (inter-personal). Although not explicitly included in any MHL definition, help-seeking behaviour was also assessed as the term is sometimes confused with help-seeking intentions. Domains were coded at an item level due to many articles presenting this form of data (e.g. % of sample that answered each item correctly as opposed to a scale mean). Frequencies and percentages were produced across all articles and by study design and definition usage.

### Assessment of measures

An assessment of all MHL related measurement tools was conducted in order to assess methodological homogeneity across articles, and whether there was evidence that the measures were psychometrically valid for adolescent samples. In order to present instruments with the most comprehensive psychometric assessments, measures were coded based on whether an article existed with the primary aim of establishing its psychometric properties with an adolescent sample.

## Results

### Article selection and characteristics

**Fig. 2:**
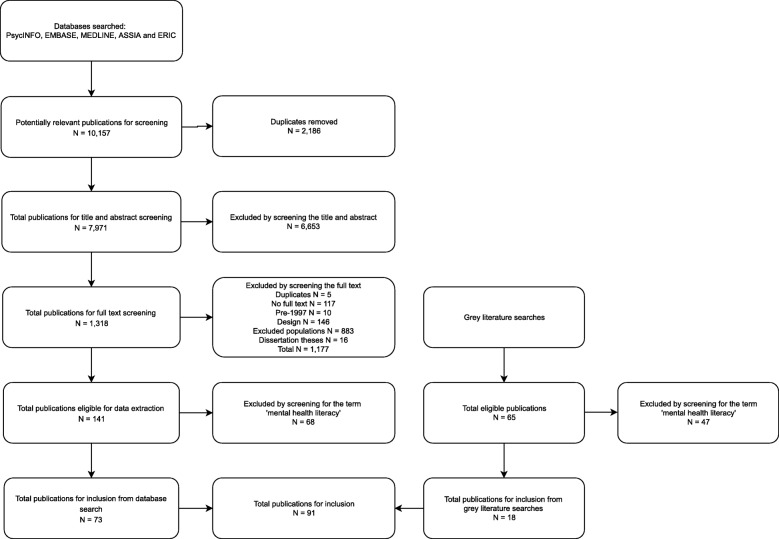
PRISMA Flowchart of Included Studies

In total, 206 articles were identified that presented extractable adolescent data on at least one MHL domain. Of these, 91 articles (44%) used the term ‘mental health literacy’. Those that did not use the term (*N* = 115, 56%), were therefore not perceived to have intended to explicitly measure the construct and were not included beyond this point. (see Fig. 2. for a PRISMA flowchart of articles, Additional File 2. for the full set of coded articles, and Additional File 3. for the reference list of included articles).

### Synthesised findings

#### Design, context and aims

**Fig. 3:**
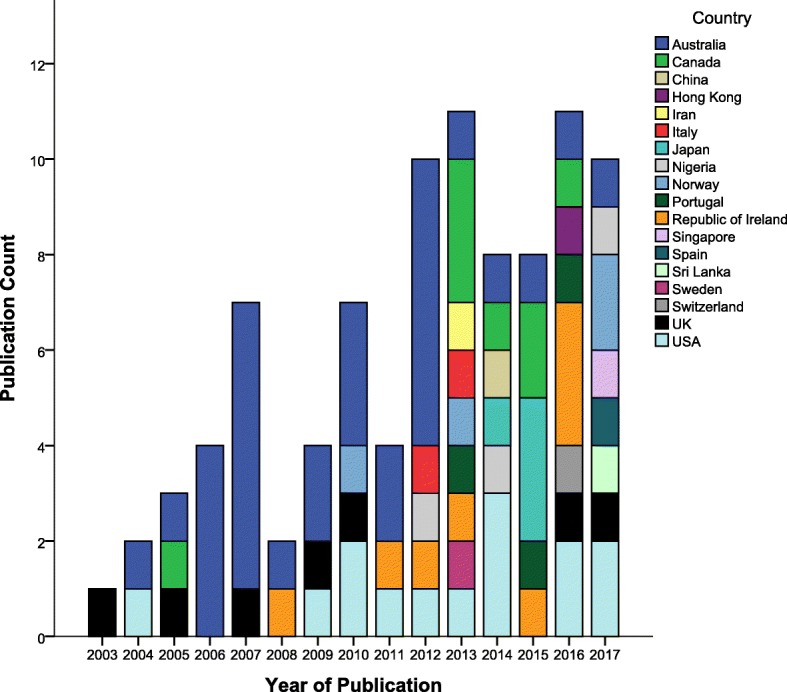
Publication Count by Year and Country

Figure 3 shows the number of publications by year and country. Australian research dominated the field up until 2013, at which point there was an increase in research being published globally. Australia (34%), USA (15%), Canada (9%), Republic of Ireland (9%) and the UK (8%) have published the majority of research between 2003 and 2017.

Table 1 presents a summary of articles’ study design, context and primary aim. The majority of articles reported on school-based studies. Articles with the primary aim of describing levels of MHL also included variables such as age, school year, gender, education, socio-economic variables, occupation, urbanicity, mental health status and previous mental health service use.

**Table 1:** Frequency and Percentage of Articles’ Study Design, Context and Primary Aim

	**Study Design**	
	**Population Study**	**Intervention Study**
	**58 (64%)**	**33 (36%)**
**Study Context**		
School-based	41 (71%)	31 (94%)
**Primary Aim**		
Scale development and/or validation	4 (7%)	–
Describe levels of MHL	39 (67%)	–
Explore possible predictors of mental illness stigma	4 (7%)	–
Explore possible predictors of help-seeking attitudes and intentions	6 (10%)	–
Explore relationship between MHL domains	5 (9%)	–
Intervention evaluation i.e. assessing the impact of an intervention	–	25 (76%)
Intervention baseline study i.e. describe level of MHL, explore predictors of specific domains or relationship between MHL domains	–	8 (24%)

*Note:* For population and intervention study design, % out of 91, for study context and primary aim, % out of number of population and intervention-based articles i.e. 58 and 33 respectively

#### Conceptualisation

Of the 91 articles that used the term ‘mental health literacy’, only 41 (45%) defined it. The most common definition, presented by 29 out of 41 (71%) articles, was that coined by Jorm and colleagues [12]. A further 3 articles (7%) used a simplified or adapted version of this definition [47–49]. Four articles (10%) defined MHL as related to knowledge only (e.g. *‘knowledge of mental health problems as well as the sources of help available’*; ( [50] pp. 485)*.* The full list of MHL domains presented by Jorm and colleagues [13], was included in over a third (*N* = 14, 34%) of articles that defined the term. However, there was some variation. For example, very few of these articles (*N* = 2, 14%) referred to different types of psychological distress as well as mental disorders when presenting the recognition domain. Furthermore, in most cases (*N* = 11, 79%), ‘knowledge and beliefs’ was replaced with ‘knowledge’ only, for domains relating to causes and risk factors, self-help strategies and professional help available.

A small number of articles that defined MHL (*N* = 5, 12%) presented Jorm’s additional domains relating to mental health first aid skills and advocacy [14]. Some articles (*N* = 4, 10%) provided examples of specific MHL domains, namely recognition of mental disorders and knowledge and beliefs about appropriate help-seeking and treatment, as opposed to presenting a comprehensive list. An emerging group of articles (*N* = 5, 12%) either acknowledged mental health promotion as a component of MHL or presented Kutcher and colleagues’ four broad domains including *‘understanding how to obtain and maintain good mental health’* ( [15] pp 155).

Regardless of whether a definition was provided, approximately one third of identified articles (*N* = 31, 34%) referred to MHL as a construct separate to mental illness stigma, with some suggesting that MHL predicts stigma. For example, articles described the measurement of these constructs as separate (e.g. *‘All respondents were then asked a series of questions that assessed sociodemographic characteristics, mental health literacy, stigma …’;* ([51] pp. 941), and referred to or presented a relationship between the two constructs (e.g. *‘Participants with higher MHL displayed more negative attitudes to mental illness’*; ( [52] pp. 100)*.* There were also instances where articles presented MHL as a predictor of help-seeking intentions and attitudes (e.g. *‘Studies indicate that in general, mental health literacy improves help seeking attitudes’*; [53] (pp. 2), or used the term MHL to refer only to improved knowledge (e.g. *‘to assess the extent to which the students had learned the curriculum and developed what we called ‘depression literacy’*; ([54] pp. 230).

#### Measurement

Thirty-nine (43%) articles included items relating to general MHL. The exact terminology varied across studies e.g. mental disorder [55], mental illness [56], mental health problem [57], and mental health issue [58]. Few articles included items relating to mental health as opposed to mental ill-health. Bjørnsen et al. developed and validated a scale to assess adolescents' knowledge of how to obtain and maintain good mental health [28]. Kutcher et al. and McLuckie et al. also included an individual knowledge item that assessed an understanding of the complete mental health state (e.g. *‘People who have mental illness can at the same time have mental health’*) [59, 60].

Table 2. presents the frequency and percentage of articles that assessed different types of diagnosis-specific literacy. In line with this focus, 57 (63%) articles utilized a vignette methodology, basing questions on descriptions, stories and scenarios relating to an individual meeting diagnostic criteria for a given mental disorder. Of these articles, 12 (21%) used comparator vignettes describing individuals with physical health problems (e.g. asthma or diabetes), control characters with good academic attainment, or ‘normal issues’ or mental health problems relating to stressful life events (e.g. the death of an elderly relative or the end of a romantic relationship). Table 3. presents the frequency and percentage of articles that assessed different domains of MHL.

**Table 2:** Frequency and Percentage of Articles focusing on Diagnosis-specific Literacy

**Diagnosis-specific Focus**	**Frequency (%)**
Depressive disorders including items relating to suicidal thoughts and behaviours	67 (74%)
Psychotic disorders	42 (46%)
Anxiety disorders	28 (31%)
Social phobia	24 (86%)
Generalised anxiety disorder	6 (21%)
Panic disorder	3 (11%)
Attention deficit hyperactivity and conduct disorders	9 (10%)
Bipolar disorders	9 (10%)
Eating disorders	6 (7%)
Post-traumatic stress or related disorders	5 (5%)
Obsessive compulsive disorders	1 (1%)
Personality disorders	1 (1%)

*Note:* For social phobia, generalised anxiety disorder and panic disorder, % out of 28 articles including anxiety related items – this does not add up to 100% due to articles including more than one anxiety disorder

**Table 3:** Frequency and Percentage of Articles Assessing MHL Domains

	**Total**		**Population**		**Intervention**		**Definition**		**No Definition**	
**MHL Domain**	**N**	**%**	**N**	**%**	**N**	**%**	**N**	**%**	**N**	**%**
**Recognition**	**37**	**41%**	**28**	**48%**	**9**	**27%**	**27**	**66%**	**10**	**20%**
Recognition of a specific mental illnesses based on a vignette by providing the correct diagnostic label	31	34%								
% correct open-ended responses	20	22%								
% correct multiple-choice responses	11	12%								
Recognition of a mental illness as opposed to a physical or spiritual problem	2	2%								
Assessment of recognition using alternative methods e.g. the ability to name or recognise names of mental illnesses	4	4%								
**Knowledge**	**76**	**84%**	**48**	**83%**	**28**	**85%**	**38**	**93%**	**38**	**76%**
Correct recognition i.e. knowledge of symptoms	33	36%								
**Knowledge about mental illnesses**	**33**	**36%**	**10**	**17%**	**23**	**70%**	**15**	**37%**	**18**	**36%**
Assessed with correct and incorrect responses	21	23%								
Assessed with attitudinal responses	12	13%								
**Knowledge of prevention and promotion of mental health**	**23**	**25%**	**20**	**35%**	**3**	**9%**	**14**	**34%**	**9**	**18%**
Assessed with correct and incorrect responses	1	1%								
% of different open-ended responses	2	2%								
Assessed with attitudinal responses	20	22%								
Perceived helpfulness /intentions to use self-help strategies	15	17%								
Beliefs about preventative strategies	7	8%								
Promotion of positive mental health.	2	2%								
**Knowledge about help-seeking**	**30**	**33%**	**24**	**41%**	**6**	**18%**	**15**	**37%**	**15**	**30%**
Intra-personal knowledge about help-seeking	13	14%								
Inter-personal knowledge about help-seeking	28	31%								
Open-ended items – knowledge of help sources and actions	22	24%								
Multiple-choice items – knowledge of help-seeking actions	2	2%								
Awareness of organisations and services	6	7%								
**Mental illness stigma**	**50**	**55%**	**25**	**43%**	**25**	**76%**	**21**	**51%**	**29**	**58%**
Intra-personal stigma	9	10%								
Inter-personal stigma	50	55%								
Personal	50	55%								
Perceived	9	10%								
Attitudes and beliefs	38	42%								
Emotional reactions	13	14%								
Behavioural intentions (social distance)	25	27%								
Actual discriminatory behaviours	3	3%								
**Help-seeking beliefs**	**64**	**70%**	**46**	**79%**	**18**	**55%**	**31**	**76%**	**33**	**66%**
Intra-personal beliefs	31	34%								
Inter-personal beliefs	57	63%								
Confidence and self-perceived help-seeking knowledge	16	18%								
Perceived helpfulness of referrals, help-sources and treatments	34	37%								
Help-seeking intentions	47	52%								
Stigma towards help-seeking	5	5%								
Perceived help-seeking barriers	9	10%								
Actual help-seeking behaviours	14	15%								

*Note:* For total, all % out of 91, for population articles, all % out of 58, for intervention articles, all % out of 33, for definition provided, all % out of 41, for no definition provided, all % out of 50. Articles that assessed the ability to recognise mental illnesses using vignettes based on diagnostic criteria were also coded as measuring knowledge of symptoms

#### Assessment of measures

Measurement tools were too heterogeneous to conduct meta-analyses. As noted in Table 1, four articles (4%) had the primary aim of validating MHL related measures with adolescent samples [28, 55, 61, 62]. The scales assessed in Bjørnsen et al. and Pang et al. measured only one broad domain of MHL; knowledge of mental health promotion and mental illness stigma respectively [28, 62]. Hart et al. assessed the psychometric properties of a depression knowledge questionnaire and found a one factor general knowledge latent structure to be the best fit to the data [61]. Campos et al. aimed to provide a more comprehensive assessment of MHL, and by psychometrically assessing a pool of items, developed a 33-item tool with three latent factors: first aid skills and help seeking, knowledge/stereotypes, and self-help strategies [55]. A further 22 articles (24%), stated that some items or scales had been developed for the purpose of the study.

Thirty-nine articles (43%) stated that they based their items on Jorm and colleagues original MHL survey or later 2006 and 2011 versions [12, 63]. Furthermore, two articles (2%) included items from the Mental Health First Aid Questionnaire (MHFAQ) as detailed by Hart et al. [64]. However, there is no evidence of the validity of these surveys as whole scales, and researchers commonly selected and modified items. The Friend in Need Questionnaire, similar to Jorm and colleagues MHL survey in that it covers multiple MHL domains, was developed by Burns and Rapee to avoid leading multiple-choice answers. Instead, open-ended responses were coded in order to quantify levels of MHL [65]. Despite finding six articles (7%) that utilised a version of this questionnaire, no published validation paper was found. As part of the Adolescent Depression Awareness Programme (ADAP), an Adolescent Depression Knowledge Questionnaire (ADKQ) was developed and later validated [61]. Six articles (7%), including the validation paper, presented data using versions of the ADKQ.

Due to the multi-faceted nature of stigma, a range of measurement tools were identified across articles. The Attribution Questionnaire (AQ-27) was originally developed by Corrigan and colleagues [66, 67] along with a brief 9-item scale (r-AQ) covering the following emotional reactions: blame, anger, pity, help, dangerousness, fear, avoidance, segregation and coercion. A similar 8-item version (AQ-8-C) was also developed for children [68]. The r-AQ was adapted by Watson et al. for use with middle school aged adolescents [69], and a 5-item version was more recently validated by Pinto et al. [70]. Four articles (4%) identified in this review used variations of the r-AQ.

Link et al. developed the 5-item Social Distance Scale (SDS) [71], which was later adapted for young people [72]. This version was more recently validated with a large sample aged 15–25 [73]. Five articles (5%) cited this version of the SDS. Seven articles (8%) used variations of the World Psychiatric Association’s (WPA) social distance items [74]; however, no adolescent validation paper was found. This review also found factual and attitudinal WPA scales presented by Pinfold et al. including the Myths and Facts About Schizophrenia Questionnaire. In total, these scales, or modified versions, were used in eight articles (9%), but no validation papers were found. The Reported and Intended Behaviour Scale (RIBS) [75] was utilised in three articles (3%). This scale has been translated into Japanese and Italian, and there is evidence of its validity with adult and university student samples [76, 77]. The evidence of its validity with an adolescent sample was mixed [78].

The Depression Stigma Scale (DSS) was developed by Griffiths et al. to measure personal and perceived depression stigma [79]. Yap et al. later validated the DSS and confirmed that personal and perceived stigma were distinct constructs comprised of ‘weak-not-sick’ and ‘dangerous/unpredictable’ factors in a sample aged 15–25 [73]. Six articles (7%) utilised a version of the DSS, more commonly the items relating to personal stigma. Items from the Opinions about Mental Illness Scale (OMI) were used in two articles (2%). The original scale was cited by both [80], however, a Chinese version of the OMI has been tested for validity with a sample of secondary school students [81]. Other validated stigma scales identified included: the Attitudes Toward Serious Mental Illness Scale–Adolescent Version (ATSMI-AV) [82] (*N* = 1, 1%) and the Subjective Social Status Loss Scale [83] (*N* = 1, 1%). Measures of help-seeking attitudes and intentions were often not validated with adolescent samples. Two articles (2%) modified the General Help Seeking Questionnaire (GHSQ), previously validated for use with high school students [84]. A further two articles (2%) utilised the Self-Stigma of Seeking Help (SSOSH) scale; however, tests of its validity have only been conducted with college students [85].

## Discussion

The aims of this review were to investigate the conceptualisation and measurement of MHL in adolescent research, and scope the extent of methodological homogeneity for possible meta-analyses. The review clearly shows an increase in school-based MHL research with adolescent samples in recent years. This makes sense given that adolescence is increasingly identified as an important period for improving MHL and access to mental health services [6, 10, 11, 38]. However, the field is still dominated by research from Western, developed countries and takes a predominantly mental-ill health approach. Furthermore, numerous challenges and inconsistencies have emerged in the field over the past 20 years.

Included articles were required to use the term ‘mental health literacy’ or a diagnosis-specific equivalent. However, by first including all articles that presented data for at least one MHL domain, a large number of articles that measured domains without referring to MHL were revealed. Researchers were measuring the same constructs but providing different labels indicating problems with discriminant validity [31, 37]. It must be acknowledged that some of the articles included in the final set may have used the term without intending to measure the whole construct, and some articles were removed that measured multiple domains. For example, 16 intervention studies, previously included in a systematic literature review of the effectiveness of MHL interventions [25], were excluded from this current review because they did not use the term. Despite the exclusion of some potentially relevant data on a domain level, this criterion was considered most appropriate given one of the aims was to assess the conceptualisation of MHL.

Although under half of the articles identified defined MHL, those that did predominantly used definitions from Jorm and colleagues [12–14]. However, the various adaptations and interpretations of the original definition has clearly led to a lack of construct travelling in the field, in particular, confusion about the inclusion of beliefs and stigma related constructs as MHL domains. Furthermore, few articles referred to mental health and varying degrees of psychological distress in addition to mental illness, supporting the argument that current MHL definitions take a predominantly mental-ill health approach [16, 26].

Although an adolescent specific definition of MHL may not be necessary, definitions frequently adopted by articles in this review were developed for adults. It is important for future research to consider not only cognitive development but also the unique social structures and vulnerabilities of adolescents in the conceptualisation and assessment of MHL. Given that the definition of adolescence in the current study ranges from 10 to 19 years, it is clear that even within this age range, different developmental factors could be considered. Applying integrated models of generic health literacy to MHL that acknowledge the life course and social and environmental determinants should therefore be a future priority [86, 87].

Around a third of articles measured recognition of specific mental illnesses, with the majority using open-ended questions such as ‘*What, if anything, do you think is wrong* …’, and calculating the % of correct responses. Knowledge of mental illnesses was measured more frequently than knowledge of prevention and promotion, therefore an understanding of the complete mental health state was often neglected [27]. More research is needed to develop and validate measures that assess the ability to seek out, comprehend, appraise and apply information relating to the complete mental health state as opposed to only assessing literacy of mental disorders. By using measurement tools that predominantly focus on psychiatric labels, there is evidence to suggest that stigma could be increased [22, 23]. Given that over three quarters of intervention studies identified in this review included a measure of stigma, future research should consider the way in which mental-ill health approaches to MHL, in terms of intervention content and study measures, may influence stigma related outcomes.

It is perhaps unsurprising that the MHL field continues to be modelled on psychiatric labelling given the influence of Jorm and colleagues early work in Australia that came out of the National Health and Medical Research Council (NHMRC) Social Psychiatry Research Unit [12]. Kutcher and colleagues MHL definition also has its origins in psychiatry, but more explicitly includes understanding of mental health promotion and stigma reduction [15]. A growing body of research relating to eating disorders literacy also emphasises the need to distinguish between health promotion, prevention and early intervention initiatives in reducing the population health burden of eating-disordered behaviour and to prioritise mental health promotion programs, including those targeting stigma reduction [88–90]. This review identified an emerging group of articles that included understanding of how to obtain and maintain good mental health in their conceptualisation of MHL. However, this domain was rarely measured.

Just under half of the articles included items relating to general MHL. However, terminology was varied (e.g. mental illness, mental disorder, mental health problem, mental health issue). Leighton revealed that young people have a lack of conceptual clarity when it comes to these mental health related terms, unsurprising given the lack of consistent definitions in practice [91]. The range and subjectivity of mental health related terms reduces the meaningfulness of comparisons across MHL studies. Similarly, over half of the articles identified in this review assessed mental illness stigma, but the complexity of the construct caused heterogeneity in measurement. Intentions to seek help were the most commonly measured help-seeking belief; these findings support previous assessments of MHL measurement tools [16]. Measuring only intentions to seek help, without capturing knowledge of what help is available, will not provide a true picture of actual behaviour change. Findings also suggested that recognition and help-seeking related beliefs may be more directly associated with the MHL construct and, in line with previous literature [25], mental illness stigma was found to be a common outcome measure in MHL related interventions.

It is worth considering whether the MHL construct should continue to be stretched or whether we should accept that the multiple domains exist in their own right. For example, self-acquired knowledge and skills relating to positive psychology are being investigated, but are only just starting to emerge under the MHL construct [28, 29]. Similarly, stigma and help-seeking knowledge and beliefs are assessed as part of, and independently from, the MHL framework. Adopting a multi-construct theory approach to MHL, as suggested by Spiker and Hammer [31], would see increased focus on developing and validating measures of specific MHL domains in order to better understand the way in which these domains relate to each other.

Developing better MHL theory will help provide clear logic models and theories of change for MHL interventions aiming to improve adolescent mental health, something currently lacking in the field. Although it should be acknowledged that the aims of MHL interventions will vary based on the scope, setting and cultural context, an increased number of validated measures as well as improved MHL theory could inform decisions about the most appropriate domain to measure as the outcome i.e. is the main aim of the intervention to reduce stigma or improve help-seeking. This is particularly important for school-based evaluations of MHL interventions for which respondent burden is often a concern.

We acknowledge that there were some articles in this review that adapted adult measures and tested for face and content validity with child and adolescent mental health professionals, and internal reliability and comprehension with adolescent samples. However, in general there was a lack of psychometric work to assess factor structure of scale-based measures in this age group, with large numbers of articles presenting data on an item level. More research should be conducted like that of Campos et al., working with young people to develop and psychometrically test pools of MHL items to identify latent factors [55]. This will help to inform future conceptualisation and measurement in this age group.

Even when there was evidence of a measure’s validity for use with adolescents, many articles selected only the items relevant for their study or adapted the scale to fit the cultural context. This may, in part, be an attempt to reduce the number of items and therefore the response burden. However, adaptation to measures based on the cultural discourse around mental health aligns with school-based mental health promotion approaches that account for children’s social, cultural and political contexts [92]. This raises the important question as to whether we should be trying to test and compare mental health related knowledge across cultures, particularly given the ongoing levels of disagreement amongst mental health professions between and within countries. A previous review of cross-cultural conceptualisations of positive mental health concluded that future definitions should be inclusive and culturally sensitive, and that more work was needed to empirically validate criteria for mental health [93]. Future research should consider conducting measurement invariance on existing MHL measures across different cultures. A comparison of knowledge items and their pre-defined correct answers, could help understand cultural differences in the discourse around mental health and what it means to be mental health literate across contexts.

Given the increased political interest in mental health promotion and education [6, 38], we recommend that MHL research focuses on increasing understanding of ways to promote and maintain positive mental health, including subjective wellbeing, optimal functioning, coping and resilience [30, 94]. Examples of knowledge items with true/false responses were identified in the current review and many aligned with a biogenetic conceptualisation of mental illness. Not only could these ‘truths’ cause more negative attitudes towards individuals experiencing mental health difficulties [19], many mapped directly onto the content of interventions and therefore do not provide any evidence of adolescents’ ability to critically appraise mental health information. To enhance individual and community level critical mental health literacy, the MHL field should apply models of public health literacy that aim to increase empowerment and control over health decisions, and acknowledge the interaction between an individual’s ability and their social and contextual demands [86, 95–97]. Given that mental health is a key component of health, it is also worth questioning the usefulness of this separation moving forward; a MHL field that is playing catch up with more developed health literacy approaches could further exaggerate the existing lack of parity of esteem.

## Conclusions

MHL research with adolescent populations is on the rise, but this review has highlighted some important areas for future consideration. Increasingly stretched definitions of MHL have led to conceptual confusion and methodological inconsistency, and there is a lack of measures developed and psychometrically tested with adolescents. Furthermore, the field is still dominated by a mental-ill health approach, with limited measures assessing the promotion of positive mental health. We suggest that the MHL field moves away from assessing ‘mental disorder literacy’ and towards critical ‘mental health literacy’. A better understanding of what MHL means for adolescents is needed in order to develop reliable, valid and feasible measures that acknowledge their developmental stage and unique social and contextual demands. In conclusion, by treating MHL as a multi-construct theory, more could be understood about the mechanisms for change in improving adolescent mental health.

## Supplementary information

**Additional file 1.** example search strategy.

**Additional file 2.** full set of coded articles.

**Additional file 3.** full reference list of included articles.

## Data Availability

Link to PROSPERO review protocol included in the manuscript, example search strategy included as supplementary material.

## Abbreviation

MHL: Mental health literacy
