# Supplementary material for: A systematic literature review of existing conceptualisation and measurement of mental health literacy in adolescent research: current challenges and inconsistencies
Source: BMC Public Health. 2020 May 1;20:607. doi: 10.1186/s12889-020-08734-1 (PMC7195735; doi:10.1186/s12889-020-08734-1)
Supplement: Supplementary file 1 — Additional file 1. example search strategy. [file 12889_2020_8734_MOESM1_ESM.pdf]

Additional File 1.

Search strategy for PsycINFO (Ovid) – (1806 to November 2017):

Population

1) (adolescen* or teen* or youth* or child* or minor* or 'young people*' or 'young person*' or student* or pupil* or pediatric*).tw.

Problem

2) exp mental health/ or exp mental disorders/

3) ((mental* adj (health or disease* or disorder* or ill* or wellbeing or well-being or 'well being')) or (depress* or anxi* or schizophreni* or psych*)).m_titl.

Outcomes

4) exp health literacy/ or exp health education/ or awareness/ or stigma/ or attitudes/ or attitude to mental illness/ or intended behavior/ or health belief/ or health behavior/ or help seeking behavior/

5) (literacy or knowledge or educat* or aware* or recogni* or stigma* or antistigma* or attitud* or belief* or stereotype* or behavior* or 'help seek*' or help-seek* or 'first aid*' or first-aid* or manag* or promo* or positive* or prevent* or 'self help*' or self-help* or treatment*).m_titl.

6) 1 and (2 or 3) and 4 and 5

Exclusions

7) 6 not (nutrition* or drug* or alcohol* or substance* or tobacco* or smok* or HIV or STD or sex* or autis* or diabet* or asthma*).m_titl.

8) limit 7 to English language
