# Supplementary material for: A systematic literature review of existing conceptualisation and measurement of mental health literacy in adolescent research: current challenges and inconsistencies
Source: BMC Public Health. 2020 May 1;20:607. doi: 10.1186/s12889-020-08734-1 (PMC7195735; doi:10.1186/s12889-020-08734-1)
Supplement: Supplementary file 3 — Additional file 3. full reference list of included articles. [file 12889_2020_8734_MOESM3_ESM.docx]

**Included Articles**

1. Andersson HW, Bjørngaard JH, Kaspersen SL, Wang CEA, Skre I, Dahl T. The effects of individual factors and school environment on mental health and prejudiced attitudes among Norwegian adolescents. Soc Psychiatry Psychiatr Epidemiol. 2010;45(5):569–77. doi:10.1007/s00127-009-0099-0

2. Attygalle UR, Perera H, Jayamanne BDW. Mental health literacy in adolescents: ability to recognise problems, helpful interventions and outcomes. Child Adolesc Psychiatry Ment Health. 2017;11(38). doi:10.1186/s13034-017-0176-1

3. Beamish N, Cannan P, Fujiyama H, Matthews A, Spiranovic C, Briggs K, et al. Evaluation of an online youth ambassador program to promote mental health. Youth Stud Aust. 2011;30(2):41–7.

4. Beirne M, Mohungoo N, Buckley S. Mental health knowledge and attitudes in a transition year student group: a pilot survey. Ir J Psychol Med. 2013;30(1):67–72. doi:10.1017/ipm.2012.7

5. Bella-Awusah T, Adedokun B, Dogra N, Omigbodun O. The impact of a mental health teaching programme on rural and urban secondary school students’ perceptions of mental illness in southwest Nigeria. J Child Adolesc Ment Health. 2014;26(3):207–15. doi:10.2989/17280583.2014.922090

6. Bjørnsen HN, Eilertsen MB, Ringdal R, Espnes GA, Moksnes UK. Positive mental health literacy: development and validation of a measure among Norwegian adolescents. BMC Public Health. 2017;17(1):717. doi:10.1186/s12889-017-4733-6

7. Bowers H, Manion I, Papadopoulos D, Gauvreau E. Stigma in school-based mental health: perceptions of young people and service providers. Child Adolesc Ment Health. 2013;18(3):165–70. doi:10.1111/j.1475-3588.2012.00673.x

8. Bulanda JJ, Bruhn C, Byro-Johnson T, Zentmyer M. Addressing mental health stigma among young adolescents: evaluation of a youth-led approach. Health Soc Work. 2014;39(2):73–80. doi:10.1093/hsw/hlu008

9. Burns JR, Rapee RM. Adolescent mental health literacy: young people’s knowledge of depression and help seeking. J Adolesc. 2006;29(2):225–39. doi:10.1016/j.adolescence.2005.05.004

10. Byrne S, Swords L, Nixon E. Mental health literacy and help-giving responses in Irish adolescents. J Adolesc Res. 2015;30(4):477–500. doi:10.1177/0743558415569731

11. Calear AL, Batterham PJ, Griffiths KM, Christensen H. Generalized anxiety disorder stigma in adolescents: personal and perceived stigma levels and predictors. Stigma Heal. 2017;2(3):208–15. doi:10.1037/sah0000046

12. Calear AL, Griffiths KM, Christensen H. Personal and perceived depression stigma in Australian adolescents: magnitude and predictors. J Affect Disord. 2011;129(1–3):104–8. doi:10.1016/j.jad.2010.08.019

13. Campos L, Dias P, Palha F, Duarte A, Veiga E. Development and psychometric properties of a new questionnaire for assessing mental health literacy in young people. Univ Psychol. 2016;15(2):61–72. doi:10.111 44/Javeriana.upsy15-2.dppq

14. Caporino NE, Karver MS. The acceptability of treatments for depression to a community sample of adolescent girls. J Adolesc. 2012;35(5):1237–45. doi:10.1016/j.adolescence.2012.04.007

15. Chisholm K, Patterson P, Torgerson C, Turner E, Jenkinson D, Birchwood M. Impact of contact on adolescents’ mental health literacy and stigma: the SchoolSpace cluster randomised controlled trial. BMJ Open. 2016;6(2). doi:10.1136/bmjopen-2015-009435

16. Coles ME, Ravid A, Gibb B, George-Denn D, Bronstein LR, McLeod S. Adolescent mental health literacy: young people’s knowledge of depression and social anxiety disorder. J Adolesc Heal. 2016;58(1):57–62. doi:10.1016/j.jadohealth.2015.09.017

17. Cotton SM, Wright A, Harris MG, Jorm AF, McGorry PD. Influence of gender on mental health literacy in young Australians. Aust N Z J Psychiatry. 2006;40(9):790–6. doi:10.1080/j.1440-1614.2006.01885.x

18. D’Avanzo B, Barbato A, Erzegovesi S, Lampertico L, Rapisarda F, Valsecchi L. Formal and informal help-seeking for mental health problems. A survey of preferences of Italian students. Clin Pract Epidemiol Ment Heal. 2012;8. doi:10.2174/1745017901208010047

19. Dey M, Reavley NJ, Jorm AF. Young people’s difficulty in talking to others about mental health problems: an analysis of time trends in Switzerland. Psychiatry Res. 2016;237:159–65. doi:10.1016/j.psychres.2016.01.048

20. Dogra N, Omigbodun O, Adedokun T, Bella T, Ronzoni P, Adesokan A. Nigerian secondary school children’s knowledge of and attitudes to mental health and illness. Clin Child Psychol Psychiatry. 2012;17(3):336–53. doi:10.1177/1359104511410804

21. Dolphin L, Hennessy E. Depression stigma among adolescents in Ireland. Stigma Heal. 2016;1(3):185–200. doi:10.1037/sah0000025

22. Essau CA, Olaya B, Pasha G, Pauli R, Bray D. Iranian adolescents’ ability to recognize depression and beliefs about preventative strategies, treatments and causes of depression. J Affect Disord. 2013;149(1–3):152–9. doi:10.1016/j.jad.2013.01.016

23. Garcia-Soriano G, Roncero M. What do Spanish adolescents think about obsessive-compulsive disorder? Mental health literacy and stigma associated with symmetry/order and aggression-related symptoms. Psychiatry Res. 2017;250:193–9. doi:10.1016/j.psychres.2017.01.080

24. Greenblatt AM, Pinto MD, Higgins MK, Berg CJ. Exploring the relationships among level of contact, nature of contact, and mental illness stigma in adolescent girls. Issues Ment Health Nurs. 2016;37(1):10–8. doi:10.3109/01612840.2015.1087604

25. Haavik L, Joa I, Hatloy K, Stain HJ, Langeveld J. Help seeking for mental health problems in an adolescent population: the effect of gender. J Ment Heal. 2017:1–8. doi:10.1080/09638237.2017.1340630

26. Hart LM, Mason RJ, Kelly CM, Cvetkovski S, Jorm AF. “ teen Mental Health First Aid ”: a description of the program and an initial evaluation. Int J Ment Health Syst. 2016;10(3):1–19. doi:10.1186/s13033-016-0034-1

27. Hart SR, Kastelic EA, Wilcox HC, Beth M, Rashelle B, Kathryn JM, et al. Achieving depression literacy: the Adolescent Depression Knowledge Questionnaire ( ADKQ ). School Ment Health. 2014;(6):213–23. doi:10.1007/s12310-014-9120-1

28. Hartman LI, Michel NM, Winter A, Young RE, Flett GL, Goldberg JO. Self-stigma of mental illness in high school youth. Can J Sch Psychol. 2013;28(1):28–42. doi:10.1177/0829573512468846

29. Hernan A, Philpot B, Edmonds A, Reddy P. Healthy minds for country youth: help-seeking for depression among rural adolescents. Aust J Rural Health. 2010;18(3):118–24. doi:10.1111/j.1440-1584.2010.01136.x

30. Hess SG, Cox TS, Gonzales LC, Kastelic EA, Mink SP, Rose LE, et al. A survey of adolescents’ knowledge about depression. Arch Psychiatr Nurs. 2004;18(6):228–34. doi:10.1016/j.apnu.2004.09.005

31. Jorm AF. Australian young people’s awareness of headspace, beyondblue and other mental health organizations. Australas Psychiatry. 2009;17(6):472–774. doi:10.1080/10398560902915596

32. Jorm AF Wright A. Beliefs of young people and their parents about the effectiveness of interventions for mental disorders. Aust N Z J Psychiatry. 2007;41(8):656–66. doi:10.1080/00048670701449179

33. Jorm AF, Kelly CM, Wright A, Parslow RA, Harris MG, McGorry PD. Belief in dealing with depression alone: results from community surveys of adolescents and adults. J Affect Disord. 2006;96(1–2):59–65. doi:10.1016/j.jad.2006.05.018

34. Jorm AF, Morgan AJ, Wright A. First aid strategies that are helpful to young people developing a mental disorder: beliefs of health professionals compared to young people and parents. BMC Psychiatry. 2008;8:1–10. doi:10.1186/1471-244X-8-42

35. Jorm AF, Morgan AJ, Wright A. Actions that young people can take to prevent depression, anxiety and psychosis: beliefs of health professionals and young people. J Affect Disord. 2010;126(1–2):278–81. doi:10.1016/j.jad.2010.03.011

36. Jorm AF, Wright A, Morgan AJ. Beliefs about appropriate first aid for young people with mental disorders: findings from an Australian national survey of youth and parents. Early Interv Psychiatry. 2007;1(1):61–70. doi:10.1111/j.1751-7893.2007.00012.x

37. Jorm AF, Wright A, Morgan AJ. Where to seek help for a mental disorder? Med J Aust. 2007;187(10):556–60.

38. Kelly CM, Jorm AF. Adolescents’ intentions to offer assistance to friends with depression or conduct disorder: associations with psychopathology and psychosocial characteristics. Early Interv Psychiatry. 2007;1(2):150–6. doi:10.1111/j.1751-7893.2007.00009.x

39. Kelly CM, Jorm AF, Rodgers B. Adolescents’ responses to peers with depression or conduct disorder. Aust N Z J Psychiatry. 2006;40(1):63–66. doi:10.1111/j.1440-1614.2006.01744.x

40. Klimes-Dougan B, Yuan C, Lee S, Houri AK. Suicide preventionwith adolescents suicide prevention with adolescents. Considering potential benefits and untoward effects of public service announcements. Crisis. 2009;30(3):128–35. doi:10.1027/0227-5910.30.3.128

41. Kutcher S, Wei Y, Morgan C. Successful application of a Canadian mental health curriculum resource by usual classroom teachers in significantly and sustainably improving student mental health literacy. Can J Psychiatry. 2015;60(12):580–6. doi:10.1177/070674371506001209

42. Lai ESY, Kwok CL, Wong PWC, Fu KW, Law YW, Yip PSF. The effectiveness and sustainability of a universal school-based programme for preventing depression in Chinese adolescents: a follow-up study using quasi-experimental design. PLoS One. 2016;11(2):1–20. doi:10.1371/journal.pone.0149854

43. Lam LT. Mental health literacy and mental health status in adolescents: a population-based survey. Child Adolesc Psychiatry Ment Heal. 2014;8. doi:10.1186/1753-2000-8-26

44. Law GU, Sinclair S, Fraser N. Children’s attitudes and behavioural intentions towards a peer with symptoms of ADHD: does the addition of a diagnostic label make a difference? J Child Heal Care. 2007;11(2):98–111. doi:10.1177/1367493507076061

45. Lawlor E, Breslin JG, Renwick L, Foley S, Mulkerrin U, Kinsella A, et al. Mental health literacy among Internet users. Early Interv Psychiatry. 2008;2(4):247–55. doi:10.1111/j.1751-7893.2008.00085.x

46. Lee J, Friesen BJ, Walker JS, Colman D, Donlan WE. Youth’s help-seeking intentions for ADHD and depression: findings from a national survey. J Child Fam Stud. 2014;23(1):144–56. doi:10.1007/s10826-012-9700-3

47. Leighton S. Adolescents’ understanding of mental health problems: conceptual confusion. Jounal Public Ment Heal. 2009;8(2):4–14. doi:10.1108/17465729200900009

48. Leighton S. Using a vignette-based questionnaire to explore adolescents understanding of mental health issues. Clin Child Psychol Psychiatry. 2010;15(2):231–50. doi:10.1177/1359104509340234

49. Livingston JD, Tugwell A, Korf-Uzan K, Cianfrone M, Coniglio C. Evaluation of a campaign to improve awareness and attitudes of young people towards mental health issues. Soc Psychiatry Psychiatr Epidemiol. 2013;48(6):965–73. doi:10.1007/s00127-012-0617-3

50. Loureiro LMJ, Jorm AF, Oliveira RA, Mendes AMOC, dos Santos JCP, Rodrigues MA, et al. Mental health literacy about schizophrenia: a survey of Portuguese youth. Early Interv Psychiatry. 2015;9(3):234–41. doi:10.1111/eip.12123

51. Loureiro LM, Jorm AF, Mendes AC, Santos JC, Ferreira RO, Pedreiro AT. Mental health literacy about depression: a survey of Portuguese youth. BMC Psychiatry. 2013;13. doi:10.1186/1471-244X-13-129

52. Lubman DI, Hides L, Jorm A. Beliefs of young people and their parents about the harmfulness of alcohol, cannabis and tobacco for mental disorders. Med J Aust. 2007;187(5):266–9. doi:10.1080/00048670701449179

53. Marshall JM, Dunstan DA. Mental health literacy of Australian rural adolescents: an analysis using vignettes and short films. Aust Psychol. 2013;48(2):119–27. doi:10.1111/j.1742-9544.2011.00048.x

54. Mason RJ, Hart LM, Rossetto A, Jorm AF. Quality and predictors of adolescents’ first aid intentions and actions towards a peer with a mental health problem. Psychiatry Res. 2015;228(1):31–8. doi:10.1016/j.psychres.2015.03.036

55. Mcluckie A, Kutcher S, Wei Y, Weaver C. Sustained improvements in students’ mental health literacy with use of a mental health curriculum in Canadian schools. BMC Psychiatry. 2014;14(12):379. doi:/10.1186/s12888-014-0379-4

56. Melas P, Tartani E, Forsner T, Edhborg M, Forsell Y. Mental health literacy about depression and schizophrenia among adolescents in Sweden. Eur Psychiatry. 2013;28(7):404–11. doi:10.1016/j.eurpsy.2013.02.002

57. Milin R, Kutcher S, Lewis SP, Walker S, Wei Y, Ferrill N, et al. Impact of a mental health curriculum on knowledge and stigma among high school students: a randomized controlled trial. J Am Acad Child Adolesc Psychiatry. 2016;55(5):383–391. doi:10.1016/j.jaac.2016.02.018

58. Mond J, Marks P, Hay P, Rodgers B, Kelly C, Owen C, et al. Mental health literacy and eating-disordered behavior: beliefs of adolescent girls concerning the treatment of and treatment-seeking for bulimia nervosa. J Youth Adolesc. 2007;36(6):753–62. doi:10.1007/s10964-006-9087-9

59. O’Connor C, McNamara N, O’Hara L, McNicholas F. Eating disorder literacy and stigmatising attitudes towards anorexia, bulimia and binge eating disorder among adolescents. Adv Eat Disord. 2016;4(2):125–40. doi:10.1080/21662630.2015.1129635

60. O’Driscoll C, Heary C, Hennessy E, McKeague L. Explicit and implicit stigma towards peers with mental health problems in childhood and adolescence. J Child Psychol Psychiatry. 2012;53(10):1054–62. doi:10.1111/j.1469-7610.2012.02580.x

61. Oduguwa AO, Adedokun B, Omigbodun OO. Effect of a mental health training programme on Nigerian school pupils’ perceptions of mental illness. Child Adolesc Psychiatry Ment Health. 2017;11(1):1–10. doi:10.1186/s13034-017-0157-4

62. Oh E, Jorm AF, Wright A. Perceived helpfulness of websites for mental health information. A nationasl survey of young Australians. Soc Psychiatry Psychiatr Epidemiol. 2009;44(4):293–9. doi:10.1007/s00127-008-0443-9

63. Ojio Y, Yonehara H, Taneichi S, Yamasaki S, Ando S, Togo F, et al. Effects of school-based mental health literacy education for secondary school students to be delivered by school teachers: a preliminary study. Psychiatry Clin Neurosci. 2015;69(9):572–9. doi:10.1111/pcn.12320

64. O'Keeffe D, Turner N, Foley S, Lawlor E, Kinsella A, O'Callaghan E, et al. The relationship between mental health literacy regarding schizophrenia and psychiatric stigma in the Republic of Ireland. J Ment Heal. 2016;35(2):100-8. doi:10.3109/09638237.2015.1057327

65. Olsson DP, Kennedy MG. Mental health literacy among young people in a small US town: recognition of disorders and hypothetical helping responses. Early Interv Psychiatry. 2010;4(4):291–8. doi:10.3109/09638237.2015.1057327

66. Pang S, Liu J, Mahesh M, Chua BY, Shahwan S, Lee SP, et al. Stigma among Singaporean youth: a cross-sectional study on adolescent attitudes towards serious mental illness and social tolerance in a multiethnic population. BMJ Open. 2017;7(10):1–12. doi:10.1136/bmjopen-2017-016432

67. Patalay P, Annis J, Sharpe H, Newman R, Main D, Ragunathan T, et al. A pre-post evaluation of OpenMinds : a sustainable , peer-led mental health literacy programme in universities and secondary schools. Prev Sci. 2017;18(8):995-1005. doi:10.1007/s11121-017-0840-y

68. Perry Y, Petrie K, Buckley H, Cavanagh L, Clarke D, Winslade M, et al. Effects of a classroom-based educational resource on adolescent mental health literacy: a cluster randomised controlled trial. J Adolesc. 2014;37(7):1143–51. doi:10.1016/j.adolescence.2014.08.001

69. Pinfold V, Stuart H, Thornicroft G, Arboleda-Florez J. Working with young people: the impact of mental health awareness programmes in schools in the UK and Canada. World Psychiatry. 2005;4:48–52.

70. Pinfold V, Toulmin H, Thornicroft G, Huxley P, Farmer P, Graham T. Reducing psychiatric stigma and discrimination: evaluation of educational interventions inUK secondary schools. Br J Psychiatry. 2003;182:342–6. doi10.1192/bjp.182.4.342

71. Pinto-Foltz MD, Logsdon MC, Myers JA. Feasibility, acceptability, and initial efficacy of a knowledge-contact program to reduce mental illness stigma and improve mental health literacy in adolescents. Soc Sci Med. 2011;72(12):2011–9. doi:10.1016/j.socscimed.2011.04.006

72. Robinson J, Gook S, Yuen HP, Hughes A, Dodd S, Bapat S, et al. Depression education and identification in schools: an Australian-based study. School Ment Health. 2010;2(1):13–22. doi:10.1007/s12310-009-9022-9

73. Ruble AE, Leon PJ, Gilley-Hensley L, Hess SG, Swartz KL. Depression knowledge in high school students: effectiveness of the Adolescent Depression Awareness Program. J Affect Disord. 2013;150(3):1025–30. doi:10.1016/j.jad.2013.05.033

74. Sawyer MG, Borojevic N, Ettridge KA, Spence SH, Sheffield J, Lynch J. Do help-seeking intentions during early adolescence vary for adolescents experiencing different levels of depressive symptoms? J Adolesc Heal. 2012;50(3):236–42. doi:10.1016/j.jadohealth.2011.06.009

75. Serra M, Lai A, Buizza C, Pioli R, Preti A, Masala C, et al. Beliefs and attitudes among italian high school students toward people with severe mental disorders. J Nerv Ment Dis. 2013;201(4):311–8. doi:10.1097/NMD.0b013e318288e27f

76. Sheffield JK, Fiorenza E, Sofronoff K. Adolescents’ willingness to seek psychological help: promoting and preventing factors. J Youth Adolesc. 2004;33(6):495–507.

77. Skre I, Friborg O, Breivik C, Johnsen LI, Arnesen Y, Wang CEA. A school intervention for mental health literacy in adolescents: effects of a non-randomized cluster controlled trial. BMC Public Health. 2013;13(1):873. doi:10.1186/1471-2458-13-873

78. Swartz KL, Kastelic EA, Hess SG, Cox TS, Gonzales LC, Mink SP, et al. The effectiveness of a school-based adolescent depression education program. Heal Educ Behav. 2010;37(1):11–22. doi:10.1177/1090198107303313

79. Swartz K, Musci RJ, Beaudry MB, Heley K, Miller L, Alfes C, et al. School-based curriculum to improve depression literacy among US secondary school students: a randomized effectiveness trial. Am J Public Health. 2017;107(12):1970–6. doi:10.2105/AJPH.2017.304088

80. Swords L, Hennessy E, Heary C. Adolescents’ beliefs about sources of help for ADHD and depression. J Adolesc. 2011;34(3):485–92. doi:10.1016/j.adolescence.2010.06.002

81. Townsend L, Stuart E, Ruble A, Beaudry MB, Schweizer B, Owen M, et al. The association of school climate, depression literacy, and mental health stigma among high school students. J Sch Health. 2018;87(8):567–74. doi:10.1111/josh.12527

82. Wright A, Harris MG, Wiggers JH, Jorm AF, Cotton SM, Harrigan SM, et al. Recognition of depression and psychosis by young Australians and their beliefs about treatment.Med J Aust. 2005;183(1):18-23. doi:10.5694/j.1326-5377.2005.tb06881.x

83. Wright A, Jorm AF, Mackinnon AJ. Labels used by young people to describe mental disorders: which ones predict effective help-seeking choices? Soc Psychiatry Psychiatr Epidemiol. 2012;47(6):917–26. doi:10.1007/s00127-011-0399-z

84. Yap MBH, Reavley NJ, Jorm AF. Intentions and helpfulness beliefs about first aid responses for young people with mental disorders: findings from two Australian national surveys of youth. J Affect Disord. 2012;136(3):430–42. doi:10.1016/j.jad.2011.11.006

85. Yap MBH, Reavley N, Jorm AF. Young people’s beliefs about preventive strategies for mental disorders: findings from two Australian national surveys of youth. J Affect Disord. 2012;136(3):940–7. doi:10.1016/j.jad.2011.09.003

86. Yap MB, Reavley NJ, Jorm AF. Australian youth still have limited awareness of headspace: results from a national survey. Aust N Z J Psychiatry. 2012;46(1):28–34. doi:10.1177/0004867411427808

87. Yap MBH, Reavley NJ, Jorm AF. Young people’s beliefs about the harmfulness of alcohol, cannabis and tobacco for mental disorders: findings from two Australian national youth surveys. Addiction. 2012;107(4):838–47. doi:10.1111/j.1360-0443.2011.03732.x

88. Yoshioka K, Reavley NJ, Hart LM, Jorm AF. Recognition of mental disorders and beliefs about treatment: results from a mental health literacy survey of Japanese high school students. Int J Cult Ment Health. 2015;8(2):207–22. doi:10.1080/17542863.2014.931979

89. Yoshioka K, Reavley NJ, MacKinnon AJ, Jorm AF. Stigmatising attitudes towards people with mental disorders: results from a survey of Japanese high school students. Psychiatry Res. 2014;215(1):229–36. doi:10.1016/j.psychres.2013.10.034

90. Yoshioka K, Reavley NJ, Rossetto A, Jorm AF. Beliefs about first aid for mental disorders: results from a mental health literacy survey of Japanese high school students. Int J Cult Ment Health. 2015;8(2):223–30. doi:10.1080/17542863.2014.931980

91. Zeifman RJ, Atkey SK, Young RE, Flett GL, Hewitt PL, Goldberg JO. When ideals get in the way of self-care: perfectionism and self-stigma for seeking psychological help among high school students. Can J Sch Psychol. 2015;30(4):273–87. doi:10.1177/0829573515594372
